# Comparison of the Fractional Exhaled Nitric Oxide Levels in Adolescents at Three Schools Located Three Different Distances from a Large Steel Mill

**DOI:** 10.1155/2017/6231309

**Published:** 2017-02-06

**Authors:** Murat Acat, Yusuf Aydemir, Onur Yazıcı, Mahmut Turğut, Mustafa Çörtük, Murat Çakar, Zehra Yaşar, Sami Deniz, Erdoğan Çetinkaya

**Affiliations:** ^1^Department of Pulmonology, Karabük University, Karabük, Turkey; ^2^Department of Pulmonology, Sakarya University, Sakarya, Turkey; ^3^Department of Pulmonology, Adnan Menderes University, Aydın, Turkey; ^4^Department of Pediatrics, Karabük University, Karabük, Turkey; ^5^Department of Pediatric Surgery, Karabük University, Karabük, Turkey; ^6^Department of Pulmonology, Abant Izzet Baysal University, Bolu, Turkey; ^7^Clinic of Chest Diseases, Dr. Suat Seren Chest Diseases and Thoracic Surgery Training and Research Hospital, İzmir, Turkey

## Abstract

*Objectives.* Exposure to ambient metals and air pollutants in urban environments has been associated with impaired lung health and inflammation in the lungs. Fractional exhaled nitric oxide (FeNO) is a reliable marker of airway inflammation. In this study, we aimed to compare the FeNO levels of three schools that have different distances from iron and steel industry zone for assessing the effects of heavy metals and air pollution on their respiratory health.* Methods.* Pulmonary function test and FeNO measurements were evaluated in 387 adolescents in three schools which have different distance from plant.* Results.* FeNO levels were significantly higher in School I (*n* = 142; 18.89 ± 12.3 ppb) and School II (*n* = 131; 17.68 ± 7.7 ppb) than School III (*n* = 114; 4.28 ± 3.9 ppb). Increased FeNO concentration was related to the distance of iron and steel industry zone in young adults.* Conclusion.* The FeNO concentrations in school children were inversely proportional to the distance from the steel mill. There are needed some studies that can evaluate the safe distance and legislation must consider these findings.

## 1. Introduction

Fractional exhaled nitric oxide (FeNO) measurements are a simple, highly reproducible, noninvasive method to indicate airway inflammation and have been investigated extensively in asthma [1]. Currently, FeNO is viewed as a marker of pulmonary inflammation in asthma [2].

Various cells in the lower airways synthesise NO via the oxidation of l-arginine by NO synthase. NO modulates vasomotor and bronchomotor tone and acts as a proinflammatory mediator; thus, it may cause oxidative tissue damage. As such, exhaled NO directly reflects the proinflammatory cytokine mechanisms of central importance in the pathophysiology of asthma and allergy [3].

There is growing evidence of significant associations between FeNO levels and air pollution (*i*.*e*., particulate matter, ambient NO, and carbon monoxide) or occupational exposure (*i*.*e*., organic solvents and heavy metals) [4–7].

Considering the simplicity of the measurements, especially with portable NO analysers, FeNO can be used to screen large populations cost effectively. Hence, FeNO may serve as a subclinical inflammation marker for monitoring the environmental health effects of these contaminants [8]. The use of this biomarker in population-based epidemiological research has great potential for assessing the impact of changing real-world mixtures of ambient air pollutants on the respiratory health of children [9].

Many studies have confirmed that the dust emitted in the production of iron and steel is an important source of ambient air particulate matter in some areas [10]. The smelting process in the iron and steel industry is known to be a major source of ambient nickel, iron, vanadium, lead, copper, and zinc, and an association between the airborne concentrations of nickel, vanadium, zinc, iron, and FeNO has been reported [4]. Another study showed that the concentrations of all measured heavy metals in an iron and steel industry zone were 1–3.5 times higher than those measured in other areas [10].

Although the effects of pollutants from industrial plants are well known, less is known about how much they affect people living near the industrial plants and what safe distances are. Therefore, this study compared the FeNO levels of children at three schools located at different distances from an iron and steel industry zone to assess the effects of heavy metals and air pollution on their respiratory health.

## 2. Materials and Methods

The study was carried out in three schools located at three different distances from the Karabük Heavy Steel and Iron Plant. This factory established in 1937 is one of the country's largest industrial plants.

**Figure 1:**
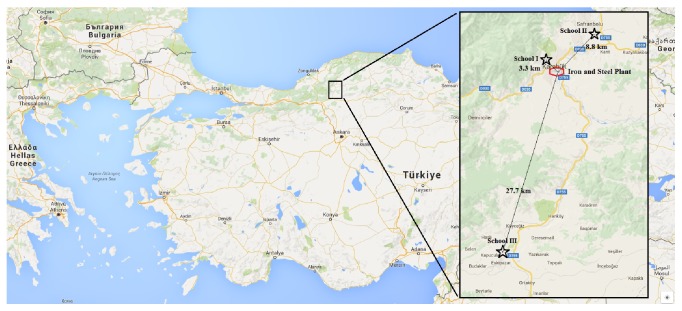
The location of the schools, according to the heavy industry area.

The annual steel production capacity is about 1.5 million tons. Schools were determined randomly among all schools in the province and named as School I, the nearest, 3.3 km, from the plant, School II, 8.8 km from the plant, and School III, the furthest, 27.7 km, from the plant (Figure 1). Address based school registration system is used in our country. Therefore, children go to the nearest school from their homes.

The study was carried out simultaneously by three different teams, using the same device. There was no highway with heavy traffic close to the schools. There was no facility or factory causing air pollution near 5 km from the schools except for School I. All students were evaluated by chest diseases specialist before participating the study. The participants underwent pulmonary function test (PFT), in accordance with the directives of the American Thoracic Society. Informed consent forms were obtained from the parents of these children. Infection, allergy symptoms, and smoking at home were questioned and those who were identified within last two weeks were excluded. If there was no allergic and atopic history, the participant was included in the study without being subjected to the allergy test. The exclusion criteria are as follows:

1. asthma, allergic rhinitis, and active pulmonary tuberculosis,
2. upper and/or lower respiratory tract infection,
3. immunologic-rheumatic disorders,
4. those that have menstrual cycles of female students,
5. smoking (active or second hand),
6. those who do not want to participate.

**Figure 2:**
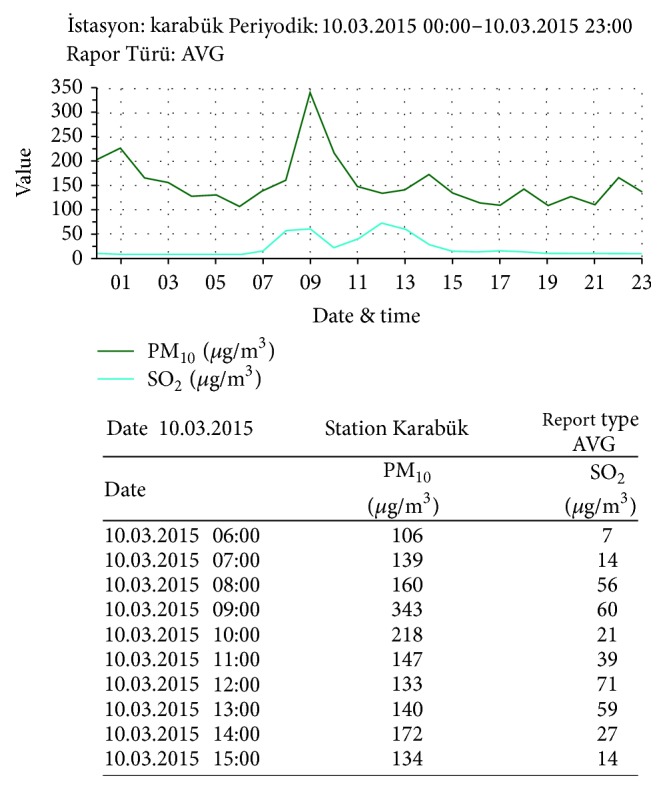
The amount of airborne PM_10_ and SO_2_.

All processes were performed before lunch and at the end of the course for avoiding the effects of foods containing nitrates, like salad greens, caffeine, consumption of alcoholic beverages, and water. All measurements were made between 12:00 and 14:00 and the wind direction was 3 km/h from the west. The amounts of airborne particulate matter (PM_10_) and SO_2_ are given in Figure 2.

FeNO was measured according to the American Thoracic Society/European Respiratory Society (ATS/ERS) guidelines [11] using a handheld device NObreath (NObreath® FeNO Monitor from Bedfont Scientific Ltd., England). An important characteristic of device is its high sensitivity down to the level of a few parts per billion (ppb) (<5 ppb). Before measuring, each child was asked to rinse their mouth with 10% NaHCO3. Three measurements were performed which were among 30 seconds relaxed tidal breathing for each child. During the measurements students were in sitting position. The measurements of FeNO plateau of at least 3 sec and 6–15 sec expiration duration were taken into consideration. Nose latch was not used during measurements. Participants who were unable to perform the practice or actual blows after a few attempts were excluded from the study. And also ethical approval and local permissions were obtained for this study.

*Statistical Analysis.* The SPSS version 21 (IBM Corporation, Armonk, NY, USA) program was used for statistical analysis. We used the independent-samples *t*-test and ANOVA for comparison of parametric data among groups. For the corrections of age, sex, and BMI we used general linear model. The limit for statistical significance was accepted as *p* < 0.05.

## 3. Results

501 students were enrolled in this study. Students who were missing the criteria and did not want to participate in the study were ruled out. FeNO measurements of the remaining 387 students were performed. The study population comprised 182 males and 205 females. The mean age of the total study population was 15.5 + 1.42 (13 to 18 years). There were significant difference in terms of age (*p* < 0,001) but there were no significant differences in body mass index (BMI) and gender between groups (resp., *p* = 0.294, *p* = 0.533). Characteristics of study population are seen in Table 1.

**Figure 3:**
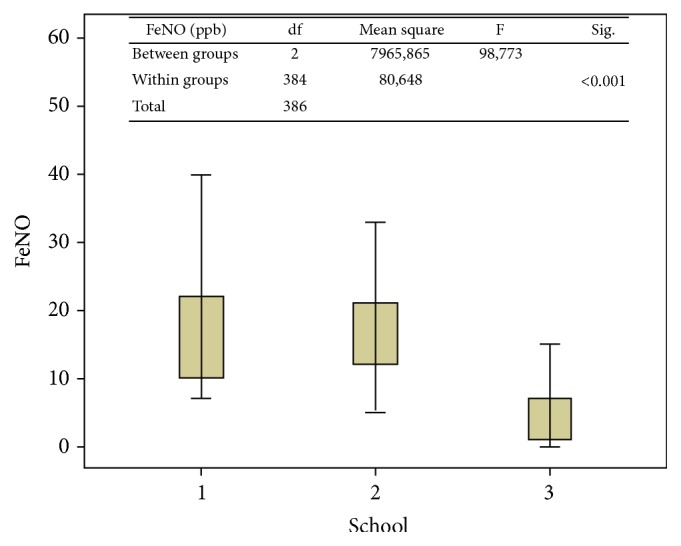
FeNO levels of three schools.

Between the groups of children, FeNO values of students at Schools I and II were significantly higher than School III (*p* < 0.001 and *p* < 0.001, resp.). There were no significant differences between School I and School II (*p* = 0.335). Also, there were statistically significant decreases in FeNO value when groups moved away from plant (*p* < 0.001). FeNO value of School III was lower fourfold than others. Results were shown in Table 2 and Figure 3.

There were no statistically significant differences in pulmonary function tests between groups. Nonetheless, predicted peak expiratory flow (PEF) values were significantly lower in the closest school to the factory (School I) as compared with Schools II and III (*p* = 0.002), even though there were no significant correlations between PEF%, FEV_1_, FVC, and FEV_1_/FVC and FeNO values (*p* = 0.316, *r* = 0.061).

## 4. Discussion

Heavy metals inhaled with atmospheric particles may adversely affect human health by accumulating in the lungs [12, 13]. There is strong evidence that children living in areas with high air pollution levels or high traffic densities have chronically increased levels of exhaled NO. One of the most important sources of heavy metals is steel plants. To our knowledge, no studies have examined the association between FeNO and the air pollution produced by these plants. The current study showed that the FeNO concentrations in school children were inversely proportional to the distance from the steel mill. The highest FeNO levels were in children attending the school nearest to the plant and lowest levels were in the school farthest from the steel plant.

Most studies of the relationship between air pollution and high FeNO levels have examined asthmatic children [14]. Although the relationship in asthmatic children is well known, fewer studies have examined healthy children. A study conducted in the Netherlands that measured FeNO and ambient air pollution levels for 7 weeks in healthy children found that the increases in the levels of various air pollutants were positively associated with the levels of FeNO [6]. In a study conducted in Seattle, ambient particulate matter less than 2.5 *μ*m in diameter (PM_2.5_) was found to be associated with exhaled NO in children with and without asthma [14]. A study conducted in healthy children aged 9–11 years in New York found a relationship between exposure to ambient metals and increased FeNO [4]. Another study performed at 25 schools in the Netherlands found a positive association between ambient PM_10_ concentrations on the day exhaled NO was measured [15]. A French study found significant positive associations between schoolyard and classroom PM_2.5_ levels and exhaled NO in children with and without asthma [8]. Similar results have been obtained in studies performed in adults [7, 16, 17].

Several studies have shown that the harmful effects of airborne pollutants are caused by oxidative stress and inflammation [18, 19]. The exposure of human lung epithelial cells to ambient fine particles in vitro showed that increased reactive oxygen species (ROS) production and inflammatory processes were initiated by the release of mediators such as IL-6, IL-8, and TNF-alpha [20]. Despite the wide variation in particle composition, evidence suggests that the secretion of cytokines by lung epithelial cells exposed in vitro to particles from different sources is regulated by common cell-signalling pathways. These findings imply that particle-mediated injury follows common pathogenic mechanisms. Apart from oxidative stress and inflammation, particle-bound materials deposited in the lungs interact directly with pulmonary cells and damage them. Soluble oxygen-transferring materials and electrophiles (such as transition metals) can cause local damage and cross the cell membranes of pulmonary epithelial cells [18].

Pulmonary function test (PFT) measures were not associated with the levels of FeNO in the current study. In a study of healthy children, Fischer et al. reported that although there was a significant relationship between ambient pollution and FeNO, there was no relationship with the PFT results [6]. Therefore, researchers have suggested that the subclinical and asymptomatic respiratory system effects of FeNO are not reflected in PFT results.

All of the abovementioned studies showed that the exhaled NO level was a useful biomonitor of individual exposure to air pollutants and to airborne particles.

Air pollution from iron- and steel-making operations has always been an environmental concern. The characterisation of heavy metals in ambient air particulate matter emissions from steel plants has been reported for many countries worldwide, including the United Kingdom, Spain, Poland, Italy, Turkey, Australia, and Korea [10]. In a study of the spatial distribution of air pollution, Kara et al. determined that an integrated pollution index was high even as far as 10 km from a heavily industrialised region [21]. In our study, the FeNO levels were significantly elevated in the students at two schools: one was 3.3 km from the plant and the other was 8.8 km.

Despite national and international laws and restrictions on the waste from industrial plants, little is known about how the spatial distribution distance of air pollutants, which is determined by geographical factors such as temperature, humidity, and wind, affects the health of children. Therefore, there are no regulations or protective mechanisms for people living close to industrial plants. Our study showed that the health of students in school almost 9 km from the plant was affected negatively. While industrial plants are often originally built outside residential areas, they become surrounded by urban areas as cities expand as a result of increasing population and urban sprawl. Few studies have examined the safe distances from industrial plants. Ours is the first study to do so for FeNO.

One limitation of our study was that we did not measure the ambient particulate matter in the schools. Because all of the measurements were performed on the same day, this did not affect our findings regarding the distance to the plant. A second limitation is that the heavy metal levels in the particles were not determined.

In summary, it is necessary to draw attention to the effects of atmospheric heavy metal pollution on people living near industrial plants. Studies need to evaluate safe distances and legislation must consider these findings.

## Figures and Tables

**Table 1:** Characteristics of patients included in the study.

	School I (3.3 km near factory) *n* = 142	School II (8.8 km near factory) *n* = 131	School III (27.7 km near factory) *n* = 114	*p *value
Age (year)	15.7 ± 1.4	14.3 ± 0.7	16.6 ± 1.1	<0.001
Sex F/M (%)	71/71 (50/50)	69/62 (52.7/47.3)	65/49 (57/43)	0.535
BMI (kg/m^2^)	20.4 ± 2.6	20.7 ± 3.3	20.9 ± 2.5	0.274
FEV_1_%	100.6 ± 12.2	100.9 ± 13.1	101.6 ± 9.7	0.795
FVC%	97.1 ± 12.2	97.3 ± 12.9	96.4 ± 12.5	0.845
FEV_1_/FVC	90.7 ± 6.2	90.8 ± 5.8	91.5 ± 5.1	0.939
PEF%	82.4 ± 19.0	89.3 ± 18.5	90.2 ± 16.4	0.002

BMI: body mass index, FEV_1_: forced expiratory volume in 1 second, FVC: forced vital capacity, and PEF: peak expiratory flow. Data showed as mean value ± SEM.

**Table 2:** The comparison of FeNO value among Schools I, II, III.

	School I (3.3 km near factory) *n* = 142	School II (8.8 km near factory) *n* = 131	School III (27.7 km near factory) *n* = 114	*p* value School I vs II	*p* value School I vs III	*p* value School II vs III
FeNO (ppb)	18.89 ± 12.3	17.68 ± 7.7	4.28 ± 3.9	0.335^*∗*^	<0.001^*∗*^	<0.001^*∗*^
				0.583^*ǂ*^	<0.001^*ǂ*^	<0.001^*ǂ*^
				0.288^§^	<0.001^§^	<0.001^§^

^*∗*^Unadjusted *p* values, ^*ǂ*^adjusted by age, and ^§^adjusted by age, sex, and BMI. Data were showed as mean ± sd.
